# The pressure-derived microvascular resistance reserve and its correlation to Doppler MRR measurement—a proof of concept study

**DOI:** 10.3389/fcvm.2024.1322161

**Published:** 2024-06-03

**Authors:** András Ágoston, Azzaya Dorj, Áron Üveges, Balázs Tar, Gábor Tamás Szabó, Judit Barta, Tibor Szűk, Michael Kest, Réka Méhész, András Komócsi, Dániel Czuriga, Benjámin Csippa, Zsolt Piróth, Emanuele Barbato, Zsolt Kőszegi

**Affiliations:** ^1^ Kálmán Laki Doctoral School of Biomedical and Clinical Sciences, University of Debrecen, Debrecen, Hungary; ^2^ Department of Cardiology, Szabolcs—Szatmár—Bereg Country Hospitals and University Teaching Hospital, Nyíregyháza, Hungary; ^3^ Division of Cardiology, Department of Cardiology, Faculty of Medicine, University of Debrecen, Debrecen, Hungary; ^4^ Heart Institute, Medical School, Pécs, Hungary; ^5^ Department of Hydrodynamic Systems, Budapest University of Technology and Economics, Budapest, Hungary; ^6^ Gottsegen National Cardiovascular Center, Budapest, Hungary; ^7^ Department of Clinical and Molecular Medicine, Sapienza University of Rome, Rome, Italy

**Keywords:** fractional flow reserve (FFR), coronary flow reserve (CFR), microvascular resistance reserve (MRR), hydrostatic pressure (HP), coronary microvascular dysfunction (CMD)

## Abstract

**Background:**

Microvascular resistance reserve (MRR) is a recently introduced specific index of coronary microcirculation. MRR calculation can utilize parameters deriving from coronary flow reserve (CFR) assessment, provided that intracoronary pressure data are also available. The previously proposed pressure-bounded CFR (CFRpb) defines the possible CFR interval on the basis of resting and hyperemic pressure gradients in the epicardial vessel, however, its correlation to the Doppler wire measurement was reported to be rather poor without the correction for hydrostatic pressure.

**Purpose:**

We aimed to determine the pressure-bounded coronary MRR interval with hydrostatic pressure correction according to the previously established equations of CFRpb adapted for the MRR concept. Furthermore, we also aimed to design a prediction model using the actual MRR value within the pressure-bounded interval and validate the results against the gold-standard Doppler wire technique.

**Methods:**

Hydrostatic pressure between the tip of the catheter and the sensor of the pressure wire was calculated by height difference measurement from a lateral angiographic view. In the derivation cohort the pressure-bounded MRR interval (between MRRpb_min_ and MRRpb_max_) was determined solely from hydrostatic pressure-corrected intracoronary pressure data. The actual MRR was calculated by simple hemodynamic equations incorporating the anatomical data of the three-dimensionally reconstructed coronary artery (MRR_p−3D_). These results were analyzed by regression analyses to find relations between the MRRpb bounds and the actual MRR_p−3D_.

**Results:**

In the derivation cohort of 23 measurements, linear regression analysis showed a tight relation between MRRpb_max_ and MRR_p−3D_ (*r*^2 ^= 0.74, *p* < 0.0001). Using this relation (MRR_p−3D_ = 1.04 + 0.51 × MRRpb_max_), the linear prediction of the MRR was tested in the validation cohort of 19 measurements against the gold standard Doppler wire technique. A significant correlation was found between the linearly predicted and the measured values (*r* = 0.54, *p* = 0.01). If the area stenosis (AS%) was included to a quadratic prediction model, the correlation was improved (*r* = 0.63, *p* = 0.004).

**Conclusions:**

The MRR can be predicted reliably to assess microvascular function by our simple model. After the correction for hydrostatic pressure error, the pressure data during routine FFR measurement provides a simultaneous physiological assessment of the macro- and microvasculature.

## Introduction

Coronary microcirculatory dysfunction (CMD) is gaining increased attention in the realm of cardiology, recognized for its critical role in the diagnosis and management of acute and chronic coronary syndromes. CMD's significance stems from its association with myocardial ischemia and its potential to cause adverse cardiovascular events. Yet, its evaluation has long been hindered by limitations in imaging techniques, with the coronary microvasculature eluding direct visualization (1–6).

Historically, the gold-standard technique for assessing microcirculation has been Doppler flow velocity. Coronary flow reserve (CFR) has become an established index to quantify coronary circulation. An abnormal CFR (less than 2) is a trusted indicator of CMD, when there is no significant obstruction in the epicardial coronary artery (7–9). The pressure-bounded CFR (CFRpb) proposed by Zimmermann et al. suggests the possibility of estimating CFR without Doppler wire using exclusively invasive intracoronary pressure data (10). Despite its potential, its correlation with the traditional Doppler-derived CFR is inconsistent, potentially due to overlooked elements like the hydrostatic pressure error (11).

Recently, De Bruyne et al. introduced the microvascular resistance reserve (MRR) combining the CFR concept with intracoronary pressure data. They proposed the continuous thermodilution technique (12) for (absolute) flow determination. Though promising, the method's complexity and associated costs have necessitated the exploration of alternative techniques.

In this study, we aim to adapt the pressure-bounded CFR concept to the MRR determination, and to fine-tune the calculations by accounting for hydrostatic pressure corrections.

Our objective is to devise a predictive model for accurate MRR values and juxtapose these predictions with the gold-standard Doppler wire measurements. This endeavor could further streamline the diagnostic process, enhancing the care provided to patients with coronary syndromes.

## Methods

### Patient population

Consecutive patients who underwent invasive coronary angiography based on clinical indications were selected from the ongoing Anatomical Assessment vs. Pullback REsting full-cycle rAtio (RFR) Measurement for Evaluation of Focal and Diffuse CoronarY Disease (“READY Registry”: NCT04857762). Specifically, those with a single intermediate-severity stenosis in a main epicardial coronary artery branch were included. Patients with acute coronary syndrome, left main stenosis, ostial stenosis, prior bypass surgery, diffuse coronary artery disease and severe renal insufficiency (estimated glomerular filtration rate <30 ml/min/1.73 m^2^) were excluded. The local ethics committee of the University of Debrecen approved the study (OGYÉI/61148/2018), which was conducted in compliance with the Declaration of Helsinki.

A total of 18 patients with indications for invasive coronary angiography were included in the derivation cohort, spanning the period from March 2022 to November 2022. The study also included pre- and post-stent data from 3 patients. In addition, one further patient was investigated in all 3 main coronary branches. Consequently, the number of subject vessels investigated amounted to 23 in the derivation cohort for establishing the relations between intracoronary pressure data and the MRR values.

Further, on the basis of the relations found, linear and quadratic predictions of the MRR (MRRpl and MRRpq) were tested in the validation cohort using 19 gold standard Doppler wire measurements from our previously published study (13).

The patient demographics both in derivation and the validation cohorts along with the investigated vessels parameters are detailed in Table 1.

**Table 1:** Clinical characteristics of the derivation and validation cohorts.

	Age (average ± SD)	Gender (male/women)	Target vessel (LAD/non-LAD)	Hypertension	DM	Dyslipidemia
Derivation cohort (18 patients/23 measurements)	64.4 ± 8.8	10/8	17/6	17	10	12
Validation cohort (16 patients/19 measurements)	59.6 ± 5.7	14/2	11/8	13	8	9
Derivation cohort vs. validation cohort:	ns*	ns**	ns**	ns**	ns**	ns**

LAD, left descending coronary artery; DM, diabetes mellitus; ns, non-significant (*p* > 0.05).

* Student's *t*-test.

** Fisher exact test.

### Coronary angiography and intracoronary pressure measurements

**Figure 1:**
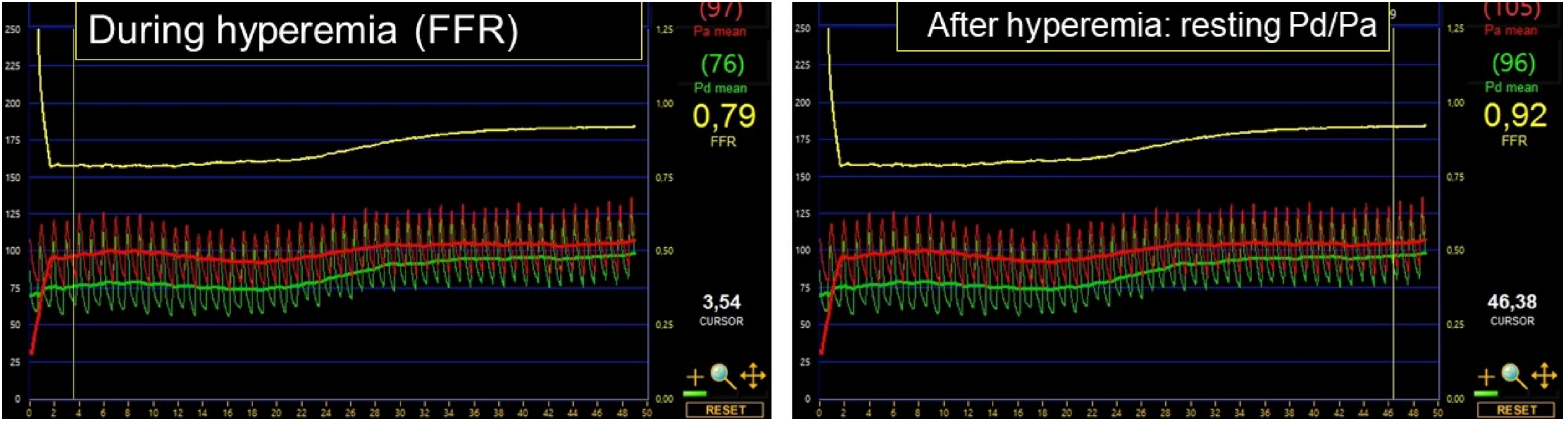
Continuous aortic (P_a_: indicated in red) and distal (P_d_: highlighted in green) pressure traces following intracoronary adenosine administration (200 µg). On the left, the marker indicates the hyperemic pressure ratio, that is, the fractional flow reserve (FFR: 0.79). On the right, the marker denotes the resting pressure ratio after the effect of adenosine subsided entirely (resting P_d_/P_a_: 0.92).

Diagnostic angiographic recordings were obtained by using the digital AXIOM Artis- x-ray device (Siemens, Munich, Germany) from the different standard projections. The intracoronary and the aortic pressure traces were recorded by the Quantien System v.1.12 (Abbott Vascular Inc., Santa Clara, CA, USA) with a pressure sensor guidewire PressureWire™ X Guidewire (Abbott Vascular Inc.). Intracoronary administration of adenosine (200 µg) was used to achieve the maximal hyperemic state. Resting and hyperemic aortic (Pa), and distal (Pd) pressure traces were recorded continuously (Figure 1).

### Determination of the hydrostatic pressure

**Figure 2:**
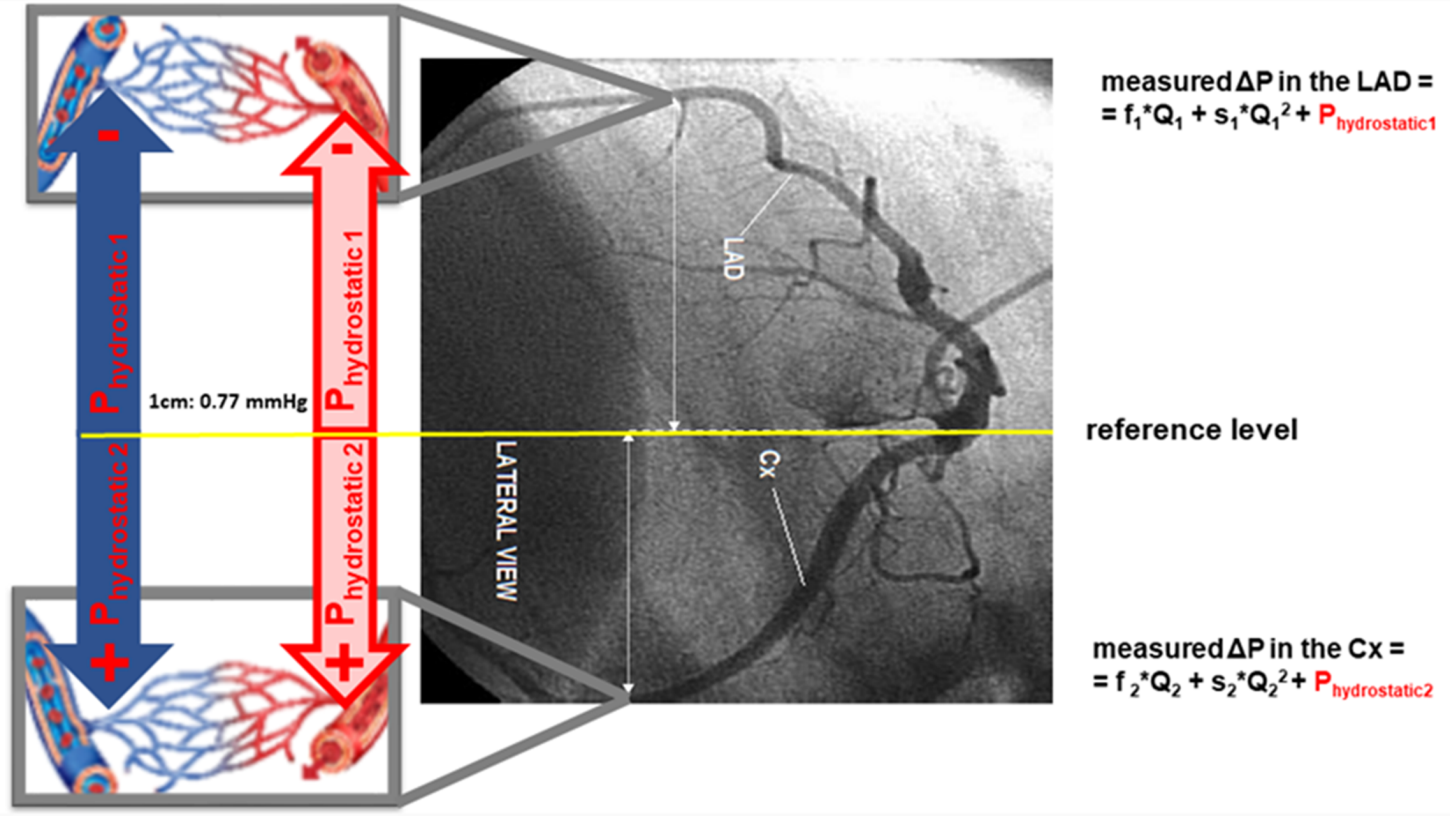
Importance of hydrostatic pressure correction in intracoronary pressure measurements taken from the supine position. The reference level of the pressure measurement system is set at the level of the distal tip of the catheter, corresponding with the aortic pressure. This position is used for the equalization with the pressure wire. When the pressure wire is advanced to the distal left anterior descending artery (LAD), a difference in height leads to a resultant decrease of hydrostatic pressure compared to the reference level. Conversely, hydrostatic pressure increases in the left circumflex artery (LCx). It is pivotal to note that these hydrostatic pressures do not contribute to the driving pressure of the blood flow, as equivalent hydrostatic pressures exist also in the venous system at the analogous level. However, when it comes to gauging epicardial pressure gradients and evaluating microvascular resistance, these hydrostatic pressure variations gain amplified significance. Even slight inaccuracies in pressure measurements may dramatically influence the upcoming calculations. ΔP, pressure drop along the target vessel; Q, volumetric flow; s, quadratic coefficient in the separation-related term; ΔP_*hydrostatic*_, hydrostatic pressure gradient; f, linear coefficient in the viscous friction pressure loss.

The hydrostatic pressure difference between the catheter tip wire sensor was calculated via height difference measurements, as depicted in Figure 2. Using the assumed blood density of 1,050 kg/m^3^, we introduced a correction factor of 0.77 mmHg of hydrostatic pressure per 1 cm of height difference in line with previous publications (7, 14, 15).

This factor was employed to adjust the measured distal pressure values during rest and hyperemia to acquire the corrected value: cPd,rest and cPd,hyp, respectively.

### Calculation of the pressure-bounded CFR and MRR (CFRpb and MRRpb)

**Figure 3:**
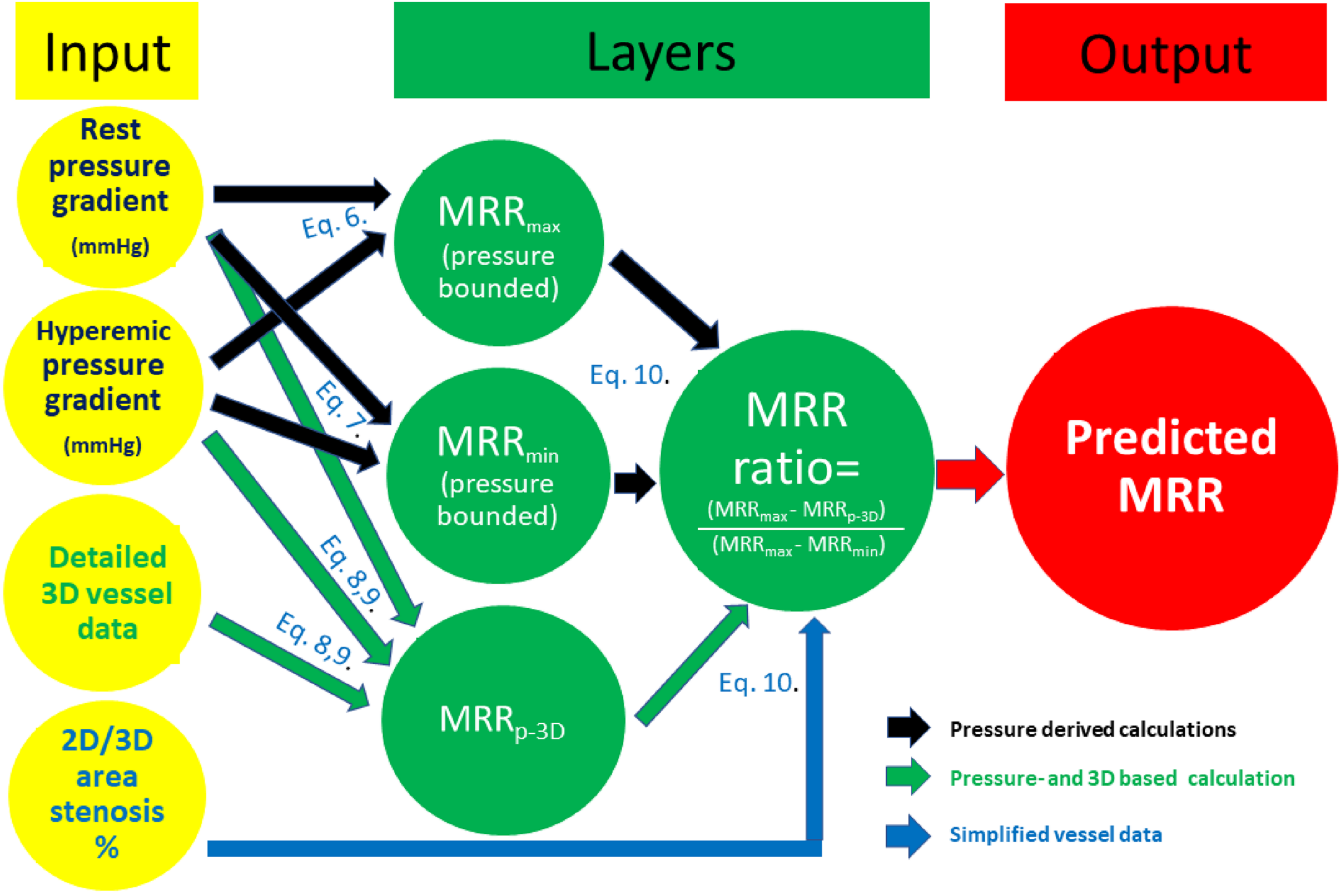
Flow chart of the calculations (see details in text).

The steps of the calculations are indicated in a flow chart of Figure 3.

The theoretical minimal and maximal bounds of CFR (CFRpb_min_ and CFRpb_max_) were determined from (hydrostatic pressure-corrected) intracoronary pressure data based on the classic equation:

(1) $$\Delta p\;=\;f\;\;\times \;Q\;+\;s\;\;\times \;Q^{2}\;+\;\Delta P_{hydrostatic}$$

where ΔP: measured pressure drop along the target vessel; f: linear coefficient in the viscous friction pressure loss; Q: volumetric flow; s: quadratic coefficient in the separation-related term; ΔP_*hydrostatic*_: hydrostatic pressure gradient.

Considering the pressure drop both in the resting state and during hyperemia, the lower bound of the CFR can be calculated by assuming the minimal CFR, as this would be the case with only a quadratic pressure drop (ΔP = s ***×*** Q^2 ^+* *Δ*P_hydrostatic_*), while the higher bound is defined as the maximal CFR, as this would be the case with only a linear pressure drop (ΔP = f ***×*** Q* + *Δ*P_hydrostatic_*) (16, 17).

Therefore:

(2) $$\sqrt{\frac{\mathrm{Hyperamic}\;\Delta p}{\mathrm{Resting}\;\Delta p}}\leq \mathrm{CFR}\leq \frac{\mathrm{Hyperamic}\;\Delta p}{\mathrm{Resting}\;\Delta p}$$

Applying hydrostatic pressure error correction, we used the following equation for our calculations: the minimum value within the pressure-bounded CFR interval (CFRpb_min_) was defined by a quadratic relation using the following equation:

(3) $$\mathrm{CFRp}b_{\min}=\sqrt{\frac{\left(\mathrm{Pa},\mathrm{hyp}-\mathrm{cPd},\mathrm{hyp}\right)}{\left(\mathrm{Pa},\mathrm{rest}-\mathrm{cPd},\mathrm{rest}\right)}}$$

The maximum value of the interval (CFRpb_max_) was calculated from a linear relation:

(4) $$\mathrm{CFRp}b_{\max}=\frac{\left(\mathrm{Pa},\mathrm{hyp}-\mathrm{cPd},\mathrm{hyp}\right)}{\left(\mathrm{Pa},\mathrm{rest}-\mathrm{cPd},\mathrm{rest}\right)}$$

The calculated CFRpb values were subsequently used to calculate the pressure-bounded MRR using the expression of the MRR formula (12):

(5) MRR=CFR/FFR×(Pa,rest/Pa,hyp)

Because FFR = Pd, hyper/Pa, hyp, by rearranging the equation

(6) MRR=CFR×(Pa,rest/Pd,hyp)

The minimum and maximum pressure-bounded MRR values were determined from the following equations:

(7) $$\mathrm{MRRp}b_{\min}\;=\;\mathrm{CFRp}b_{\min}\;\;\times \;(\mathrm{Pa},\mathrm{rest}/\mathrm{cPd},\mathrm{hyp})$$

(8) $$\mathrm{MRRp}b_{\max}\;=\;\mathrm{CFRp}b_{\max}\;\;\times \;(\mathrm{Pa},\mathrm{rest}/\mathrm{cPd},\mathrm{hyp})$$

### Three-dimensional reconstructions of coronary angiography and calculation of the actual CFR and MRR within the pressure-bounded intervals

Three-dimensional reconstructions of the vessels were carried out using the QAngio XA Research Edition 1.0 software (Medis Specials bv, Leiden). The reconstructions were based on two angiographic recordings that met specific criteria: they had to possess satisfactory visual quality and display a minimum projection angle difference of 25°. The 3D anatomical model was reconstructed to encompass the interrogated segment ranging from the coronary orifice and concluding at the distal tip of the pressure wire sensor. The software automatically extracted various geometric measurements of the lesion. These metrics comprised the mean cross-sectional diameters, lengths of the vessel segments, and both the proximal and distal reference vessel segments.

These values, along with intracoronary pressure data collected at the proximal and distal positions during the resting and hyperemic states, were combined to perform hemodynamic calculations according to Equation 9:

(9) $$\Delta \mathrm{pt}\;=\;f_{\mathrm{prox}}\;\;\times \;Q\;+\;f_{\mathrm{sten}}\;\;\times \;Q\;+\;s\;\;\times \;Q^{2}\;+\;f_{\mathrm{dist}}\;\;\times \;Q\;+\;\Delta P_{hydrostatic}$$

where Q is the volumetric flow rate; Δpt is the measured total pressure drop; f_prox_, f_sten_ and f_dist_ are the linear coefficients in the viscous pressure loss terms in the proximal (prox) stenosed (sten) and the distal (dist) segments, respectively, while s is the quadratic coefficient in the separation-related pressure loss term at the lesion.

The f and the s coefficients were defined on the basis of 3D anatomical parameters. For a detailed methodology, we refer to previous publications (13, 16–18) (the online calculation tool can be accessed at http://coronart.unideb.hu, while the Δ*P_hydrostatic_* was assessed according to the measured height differences explained in the above section.

Knowing these parameters, the *Q* values can be calculated by solving the quadratic equation:

(10) $$Q\;=\;\frac{-f\;+\;\sqrt{f^{2}\;+\;4s\;\;\times \;(\Delta P\;-\;\Delta P_{hydrostatic})}}{2s}$$

where

(11) $$f\;=\;f_{\mathrm{prox}}\;+\;f_{\mathrm{sten}}\;+\;f_{\mathrm{dist}}$$

In addition to the pressure-bounded MRR, we also determined the exact (actual) vessel-specific pressure- and 3D-derived MRR value (MRR_p−3D_) that lies within the pressure-bounded MRR interval. To achieve this, we first calculated the pressure- and 3D-derived CFR (CFR_p−3D_), which is calculated by integrating intracoronary pressure values adjusted for hydrostatic pressure with parameters derived from 3D anatomical reconstructions of the investigated vessel (13, 16–18).

MRR_p−3D_ was then calculated from the CFR_p−3D_ values using the following equation:

(12) $$\mathrm{MR}R_{p\text{-}3D}\;=\;\mathrm{CF}R_{p\text{-}3D}\;\;\times \;(\mathrm{Pa},\mathrm{rest}/\mathrm{cPd},\mathrm{hyp})$$

In order to determine the exact position of the (actual) MRR_p−3D_ within the pressure-bounded MRR interval, the MRR ratio was calculated using the following equation:

(13) $$\mathrm{MRR}\;\mathrm{ratio}\;=\;(\mathrm{MRRp}b_{\max}-\mathrm{MR}R_{p\text{-}3D})/(\mathrm{MRRp}b_{\max}-\mathrm{MRRp}b_{\min})$$

### Statistical analysis

Statistical analysis was performed using MedCalc Software 14.8.1 (MedCalc Software bvba, Ostend, Belgium).

First, a linear regression analysis was completed to acquire the relation between the maximal pressure-bounded MRR (MRRpb_max_) and the actual MRR_p−3D_ in the derivation cohort of 23 measurements (the former was calculated merely from pressure gradients, while the latter used classic hemodynamic flow equations including 3D anatomical data and measured pressure data).

Next, a quadratic regression analysis in the function of the percent area stenosis (AS%) was conducted to establish the relation between the AS% and MRR ratio.

Further, on the basis of the relations found, linear and quadratic predictions of the MRR (MRR_pl_ and MRR_pq_) were tested in the validation cohort using 19 gold standard Doppler wire measurements (13). The MRR_Doppler_ was defined as follows:

(14) $$\mathrm{MR}R_{\mathrm{Doppler}}\;=\;\mathrm{CF}R_{\mathrm{Doppler}}\;\;\times \;(\mathrm{Pa},\mathrm{rest}/\mathrm{Pd},\mathrm{hyp})$$

where

(15) $$\mathrm{CF}R_{\mathrm{Doppler}}\;=\;\mathrm{AP}V_{\mathrm{hyper}}/\mathrm{AP}V_{\mathrm{rest}}$$

and APV_hyper_ and APV_rest_: average peak velocity measured by the Doppler sensor distally to the lesion during hyperemia and in basal state, respectively.

The predicted results were compared to MRR_Doppler_ by the Pearson correlation analysis (Figure 3). The correlation between the linearly predicted MRR (MRR_pl_) and MRR_Doppler_ was assessed for all cases, and we also analyzed the correlation in the subgroup of non-ischemic FFR cases.

Additionally, the agreement between both the MRR_pl_ and the MRR_pq_ against the MRR_Doppler_ values were visualized with Bland-Altman plots.

## Results

**Figure 4:**
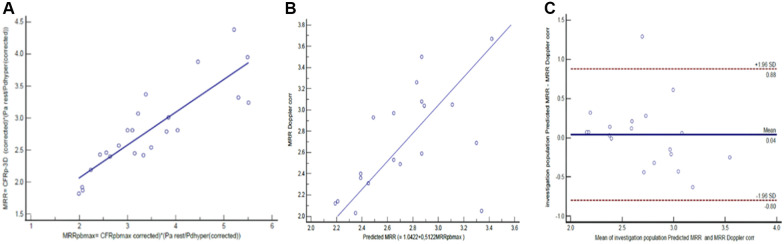
The linear relation between MRRpb_max_ and MRR_p−3D_ (**A**) in the derivation cohort (*n* = 23), the correlation (**B**) and agreement (**C**) between linear prediction for MRR (MRR_pl_) and the MRR_Doppler_ in the validation cohort (*n* = 19) (see details in text).

In the derivation cohort, the linear regression analysis yielded a strong relation between the actual value of MRR determined by the hemodynamic calculation (using both the measured intracoronary pressure data and the 3D anatomical results derived from the angiography: MRR_p−3D_) and the pressure-bounded maximal MRR (MRRpb_max_) (*r*^2 ^= 0.7436, *p* < 0.0001) (Figure 4A). The resulting linear equation (MRR_p−3D _= 1.0422 + 0.5122 × MRRpb_max_) was able to predict the Doppler-derived MRR from the MRRpb_max_ in the validation cohort. This prediction resulted in a significant correlation (*r* = 0.5418, *p* = 0.0166) between MRR_Doppler_ and the linearly predicted MRR (MRR_pl_) in the validation (test) population (Figure 4B).

The Bland-Altman analysis revealed a mean difference of 0.04 (±1.96 SD: 0.88, −0.8) between the MRR_Doppler_ and MRR_p_ values (Figure 4C).

**Figure 5:**
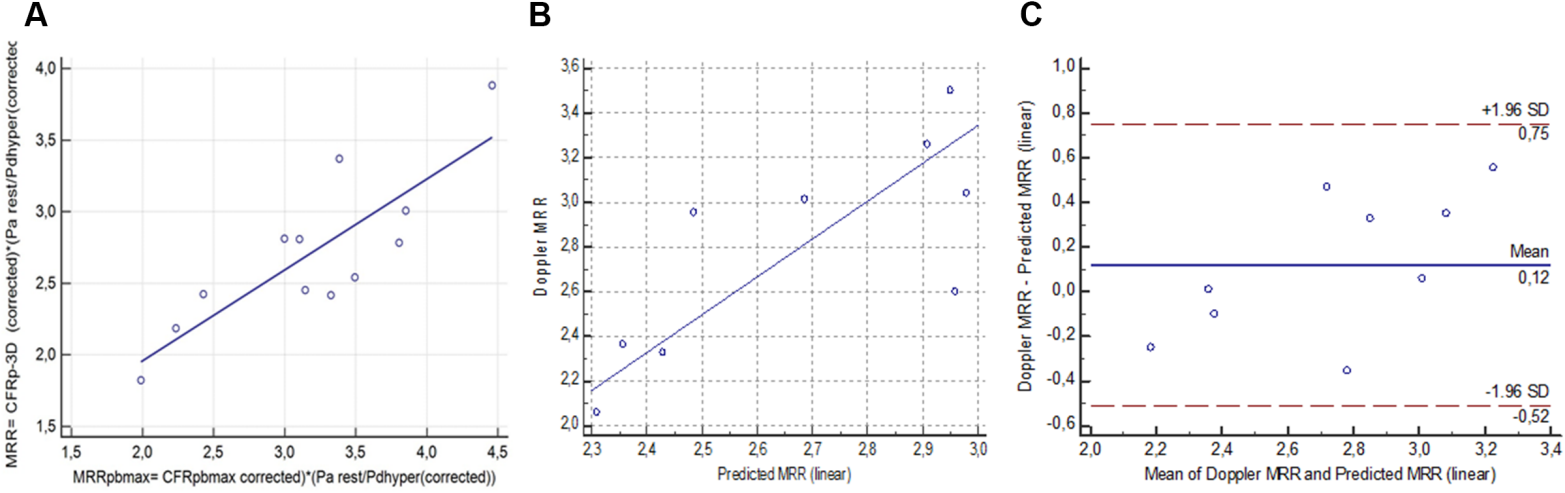
The linear relation between MRRpb_max_ and MRR_p−3D_ in the non-ischemic FFR subgroup (FFR >0.8); *r*^2 ^= 0.69, *p* = 0.0008 (**A**) in the derivation cohort (*n* = 12), the correlation (**B**) and agreement (**C**) between linear prediction for MRR and the Doppler MRR in the test population (*n* = 9). (MRR, microvascular resistance reserve; FFR, fractional flow reserve) (see details in text).

In the derivation cohort, there were 12 measurements with FFR values above 0.80. In these cases, the linear regression analysis also yielded a significant relation between the actual value of MRR_p−3D_ and the pressure-bounded maximal MRR (MRRpb_max_) (*r*^2 ^= 0.69, *p* = 0.008) (Figure 5A). The resulting linear equation (MRR_p−3D _= 0.69 + 0.64 × MRRpb_max_) led to a fair prediction of the Doppler-derived MRR from MRRpb_max_ in 9 cases from the subgroup of the validation cohort with non-ischemic FFR values. This linear prediction resulted in a good correlation (*r* = 0.76, *p* = 0.018) between MRR_Doppler_ and the pressure-derived MRR_p_ in the test population (Figure 5B). The Bland-Altman analysis revealed a mean difference of 0.12 (±1.96 SD: 0.75, −0.52) between MRR_Doppler_ and MRR_pl_ values (Figure 5C).

**Figure 6:**
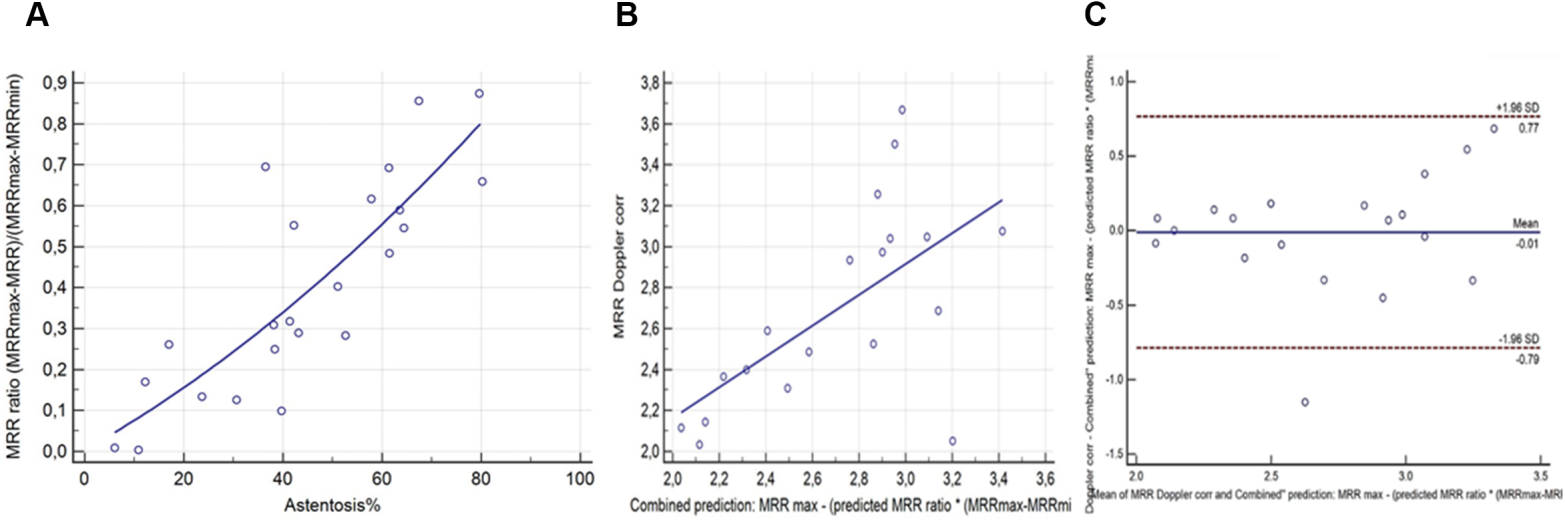
The quadratic relation between AS% and the MRR ratio. (**A**) in the derivation cohort (*n* = 23), the correlation (**B**) and agreement (**C**) between quadratic combined prediction for MRR (MRR_pq_) and the MMR_Doppler_ in the test cohort (*n* = 19).

To investigate the correlation between the position of the actual MRR_p−3D_ values inside the pressure bounded interval (MRR ratio) and the AS% defined by 3D quantitative coronary angiography, a quadratic regression analysis was performed in the derivation cohort (*y* = 0.004717 + 0.006787x + 0.00003998 × 2, *r*^2 ^= 0.6963, *p* < 0.0001) (Figure 6A).

Using this quadratic regression for the combined (incorporating both pressure and area stenosis data) prediction of the MRR_Doppler_ resulted in a stronger correlation between MRR_pq_ and the MRR_Doppler_ (*r* = 0.63, *p* = 0.004) in the test cohort of 19 measurements, than the pressure only linear MRR_pl_ prediction (Figure 6B).

Further, the Bland-Altman analysis showed reduced mean differences between the MRR_pq_ and the MRR_Doppler_ values of −0.01 (±1.96 SD: 0.77, −0.79) (Figure 6C).

The frequency of the CMD in our population based on MRRp-3D <2.5 criteria was 8/18 (44%); among them 5/8 (63%) had diabetes mellitus.

## Discussion

The pressure-bounded CFR (CFRpb) proposed by Zimmermann et al. suggests the possibility of estimating CFR without Doppler wire using exclusively invasive intracoronary pressure data (10). Despite its potential, its correlation with the traditional Doppler-derived CFR is inconsistent, potentially due to overlooked elements like the hydrostatic pressure error (11).

The functional assessment of coronary artery disease often requires invasive intracoronary pressure measurement to determine the fractional flow reserve (FFR). Alongside the assessment of epicardial coronary arteries, there is an increasing recognition of microvascular function as a crucial aspect in the diagnosis and management of both acute and chronic coronary syndromes. Therefore, characterizing the microcirculation holds significant clinical implications, while the invasive diagnosis of coronary CMD is becoming an essential tool for interventional cardiologists (1–3). An important recent advancement in the field was the recognition of CMD as a diagnosis by the International Statistical Classification of Diseases and Related Health Problems 10th revision (ICD-10), which has generated a novel ICD-10-CM code (in effect from October 1, 2023).

In the catheterization laboratory, CMD can be identified by invasive measurement of coronary blood flow or microvascular resistance. Coronary flow reserve (CFR) provides a quantitative and comprehensive evaluation of coronary circulation, reflecting disease processes affecting both the epicardial arteries and the microcirculation. CFR is conventionally assessed by the bolus thermodilution technique or reference-standard Doppler flow velocity. However, the bolus thermodilution method comes with several limitations related to the variability of the detected mean transit time due to the variable power of the manual injection of the intracoronary saline injection as well as the variable timing of the injection within the heart cycle. The transit time is also dependent significantly on the distance of the sensor from the tip of the catheter. On the other hand, good quality Doppler measurement of coronary flow velocity is technically quite difficult to achieve; and, it is not routinely used in clinical practice (4–9).

The pressure-bounded CFR (CFRpb) provides lower and upper CFR bounds based on the relationship between the flow and pressure drop across the stenosis. (The actual value of CFRpb is constrained within a range defined by a minimal and maximal value or bound). The lower bound corresponds to instances where the pressure drop across a stenosis arises solely from separation losses, while the upper bound is determined by a pressure drop arising exclusively from friction losses. CFRpb was defined as abnormal when both its upper and lower bounds were <2, and was considered normal when both bounds were ≥2. CFRpb was considered indeterminate in all other cases (10). However, a poor diagnostic agreement was reported between Doppler flow-derived CFR and CFRpb (11). We propose that the poor correlation between CFRpb and its flow-based counterpart may be due to the omission of the hydrostatic pressure error in the CFRpb calculations.

Disregarding the impact of hydrostatic pressure is an often neglected pitfall during intracoronary pressure measurements. Variations in hydrostatic pressure occur in the supine position due to the height difference between the coronary vessel orifice and the pressure sensor at the distal portion of the vessel. This pressure disparity may significantly modify the measured distal coronary pressures across different coronary segments, depending on the horizontal position of the vessel relative to the distal end of the catheter (Figure 2) (14, 15, 19).

In 2021, De Bruyne et al. utilized the continuous thermodilution technique to derive the MRR, a microvasculature-specific index that relies on quantitative absolute coronary flow values. Continuous thermodilution involves the use of a dedicated monorail infusion catheter to administer a low- and a high-rate continuous saline infusion, the latter inducing a physiologic state of maximal hyperemia, which eliminates the need for pharmacological vasodilation (12). This technique allows for the calculation of volumetric absolute coronary flow (Q), measured in milliliters/second. The true resting microvascular resistance (R_µ, rest_) can then be calculated from the ratio of resting aortic pressure and the absolute flow, while the hyperemic microvascular resistance (R_µ, hyper_) can be calculated from the ratio of distal pressure and absolute flow measured during high-rate saline-induce maximal hyperemia. Consequently, MRR can be calculated from the ratio of R_µ, rest,_ and R_µ, hyper_, and can be expressed more generally by Equation 9 (MRR = CFR × (Pa_rest_/Pd_hyp_) (12). MRR demonstrated a significant correlation with intracoronary Doppler flow measurements obtained concurrently. However, the inherent intricacy of the MRR technique, combined with its dependency on specialized equipment, has paved the way for the development of alternative methodologies.

The general concept of MRR can be applied to any method that measures flow or its surrogates, provided that resting and hyperemic pressure values can also be obtained or estimated. In this study, we adapted the MRR concept to the previously established equations of CFRpb to determine the pressure-bounded coronary MRR interval. Distal pressure measurements used in the calculations were corrected for variations in the hydrostatic pressure as well.

**Figure 7:**
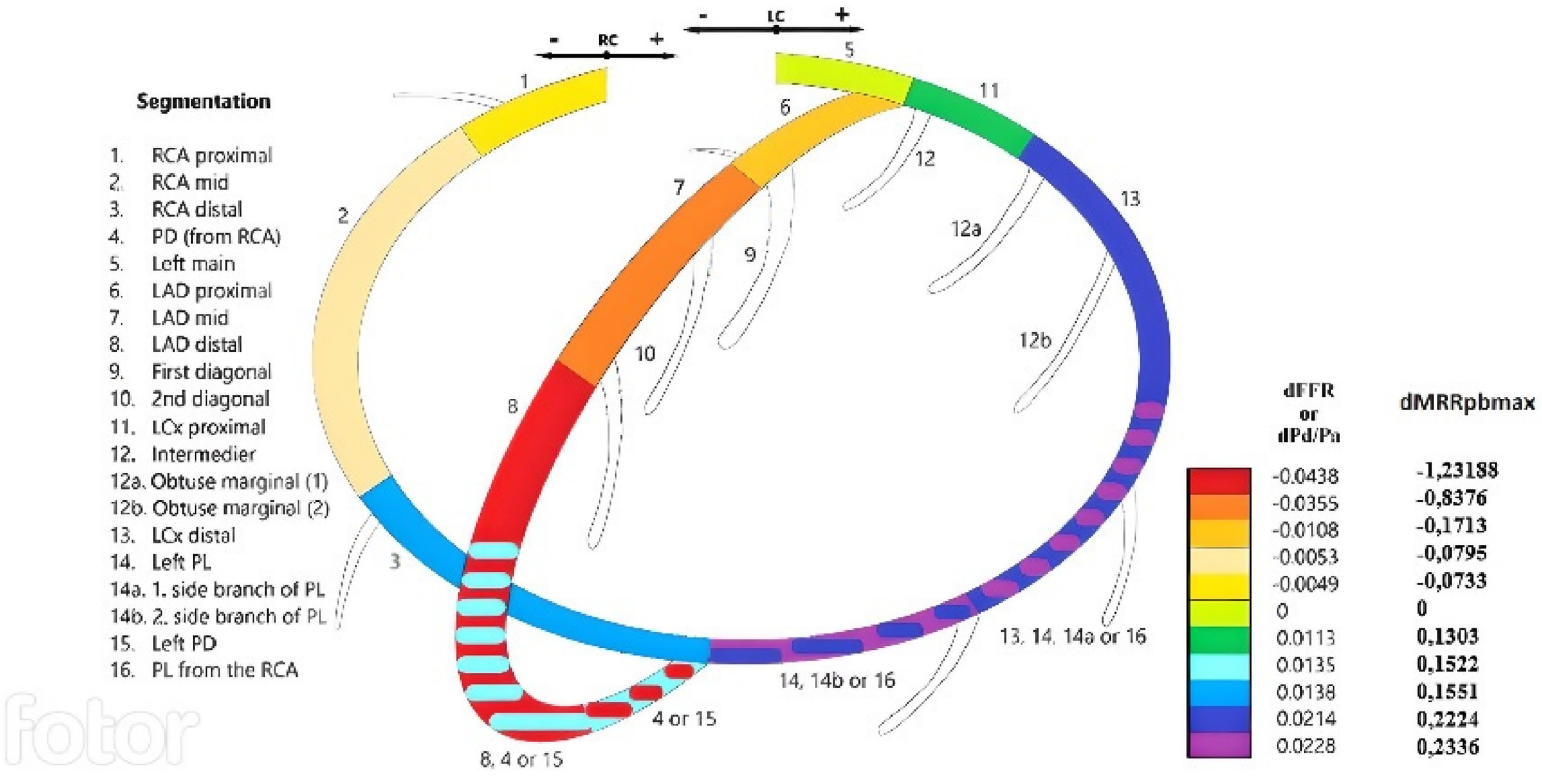
The mean change in the maximal MRR_pb_ caused by hydrostatic pressure offset in various coronary segments (right column). A modified color-coded version of the coronary segmentation defined by the Syntax scoring system (left panel) (adapted with permission from Üveges et al. (14), licensed under CC BY 4.0, https://doi.org/10.1007/s10554-020-01971-w). On this scheme, the change in fractional flow reserve and resting distal/aortic pressure values (dFFR and dPd/Pa) (first column) due to hydrostatic pressure are indicated for each epicardial segment according to the type of individual coronary circulation.

In previous publications, variations in hydrostatic pressure gradients leading to discernible differences in resting Pd/Pa and FFR values within specific coronary segments have been demonstrated (14, 15, 19). These differences are contingent upon the vertical positioning of the vessel in relation to the coronary orifice. In the context of maximal pressure-bounded MRR, the magnitude of the discrepancy is significantly amplified. In a hypothetical scenario involving a patient with aortic pressure of 100 mmHg and borderline coronary stenosis (Pd/Pa: 0.91, FFR: 0.8), we performed calculations to assess the disparities in MRRpb_max_ values with and without accounting for the hydrostatic pressure offset. The findings revealed that this discrepancy could result in a substantial, up to 123%, variation in the calculated maximal pressure-bounded MRR value, depending on whether or not hydrostatic pressure correction was applied (Figure 7).

Recently, the MRR emerged as a distinct index for microcirculation. Notably, its formulation is free from the influences of epicardial resistance, myocardial mass, autoregulation and the aortic pressure. Our introduction of the pressure-bounded MRR methodology facilitates a direct appraisal of potential MRR values using pressure recordings taken during FFR measurements. This is achieved without resorting to thermodilution techniques. By using the pressure-bounded approach, a range of maximal and minimal MRR values is determined, within which the actual MRR_p−3D_ may be found. The degree of stenosis (AS%) determines the actual MRR value to vary within this interval. In cases of mild, non-flow limiting lesions, the actual MRR_p−3D_ values tend to align more closely with the pressure-bounded maximal MRR values (MRRpb_max_). This is attributed to the fact that such lesions primarily result in a pressure loss that is linearly proportioned to the linear flow as quantified in Equation 1. Indeed, there was a noteworthy linear relation between the MRRpb_max_ and the actual MRR_p−3D_ among the derivation cohort in our study.

In contrast, in the presence of tight lesions, there is a more pronounced, quadratic pressure drop owing to the phenomenon of flow separation. As a consequence, the actual MRR_p−3D_ value tends to be more closely to the pressure-bounded MRR minimal value. Thus, using MRRpb_max_ as a predictive measure for MRR is suitable only for mild lesions, accompanied by non-ischemic FFR. However, for patients exhibiting more severe stenoses, incorporating the degree of area stenosis becomes crucial in making an accurate prediction of MRR. This consideration was demonstrated in our quadratic regression analysis between the area stenosis and the MRR ratio, and the estimation of the quadratic component of the pressure drop seems to be mandatory to be incorporated into the calculations. In our approach, the 3D quantitation of the AS% provided an indirect estimation of the effect of a flow separation-related pressure loss component on the actual position of the MRR within the theoretical pressure-bounded interval. Further, it seems that the assessment of the area stenosis could be simplified by a properly selected 2D angiographic view measurement, and therefore the 3D angiography reconstruction may be avoided (20).

On the other hand, in cases with non-ischemic FFR values, one does not expect significant flow separations, therefore the *s* value is negligible, and the linear relation between the maximal pressure-bounded MRRpb_max_ and the actual MRR may form an appropriate basis for the linear prediction of MRR, even without stenosis quantitation.

The above hypotheses seemed to be proved in our study, where the relation between the pressure bounds of the MRR interval and the calculated actual MRR values were searched in a derivation cohort. The founded relations were used to predict the MRR in a completely different validation cohort with Doppler-derived MRR values. However, rigorous validation within more expansive patient cohorts is warranted to establish clinical relevance and accuracy.

In the 2019 European Society of Cardiology (ESC) guidelines for Chronic Coronary Syndromes (CCS), a Class IIA recommendation emphasized the utility of invasive guidewire-based pressure and flow measurements for diagnosing angina rooted in the microcirculation. This diagnostic approach is particularly highlighted for patients who persistently manifest symptoms and either show angiographically normal coronary arteries or moderate stenoses with a non-ischemic FFR (21). As our presented methodology allows a feasible evaluation of CMD during routine invasive FFR measurement, it may be incorporated into the invasive physiological work-up. If FFR is preserved, CFR and MRR values shall be assessed using simple calculation methods to establish the diagnosis of CMD. A more accessible diagnostic procedure may pave the way for broader implementation of targeted therapies. When microvascular disease is identified, a range of specific treatments becomes available. These include lifestyle changes, β-blockers, angiotensin-converting enzyme inhibitors, and statins, all aimed at effectively managing the condition (22).

## Limitations

One of the main limitation of our proof of concept study is the small sample size; however, we think that the archived statistically significant agreement with the gold standard Doppler method looks promising.

Our derivation cohort was restricted to patients with chronic coronary syndrome with a single intermediate-severity stenosis in a main epicardial coronary artery branch. Patients with acute coronary syndrome, left main stenosis, ostial stenosis, prior bypass surgery, diffuse coronary artery disease were excluded, therefore it is quite obvious that our model can be less performing in an all comer patient population.

In our endeavor to develop a predictive model for MRR, we utilized intracoronary pressure data and AS% observed during coronary angiography, gleaning insights from a designated derivation cohort. Subsequent validation was undertaken using Doppler measurements in a separate test cohort. While this methodology resonates with facets of machine learning, it uniquely hinges on predefined hemodynamic equations to bridge the gap between input parameters and expected outcomes. Given the sample size of our study, an exhaustive machine learning analysis was not feasible, but the potential for such a methodological approach remains tangible with a more extensive patient dataset and broader input parameters. Notably, the precision of intracoronary pressure measurements presents inherent challenges; trivial inaccuracies may precipitate significant deviations in MRR determinations, especially in scenarios marked by low resting pressure gradients. To mitigate potential errors, meticulous efforts were invested in ensuring accurate pressure trace acquisitions. Moreover, the positioning of instruments was stringently monitored to preclude any wedging impacts, and periodic checks were made for pressure signal drifts. In instances of significant discrepancies, procedures were reiterated, while minor variations were methodically adjusted. Furthermore, we considered the frequently underestimated hydrostatic pressure error, bolstering the robustness of our calculations. This rigorous approach enabled us to achieve reliable predictions even in situations with inconspicuous pressure gradients.

## Conclusions

The prediction of MRR from a pressure-derived measurement with hydrostatic pressure correction is a simple, yet comprehensive method for assessing CMD. This method eliminates the need for using a Doppler wire or the thermodilution procedure and can be applied across all clinically indicated invasive measurements of FFR. Including the area stenosis assessment from a properly selected single angiographic view, the 3D coronary reconstruction may also be obviated. Consequently, this method facilitates both macro- and microvascular physiological assessment and may be performed straightforwardly, thereby effectively supporting the clinical decision for selecting an individually tailored therapy. If larger scale studies—preferentially with continuous thermodilution MRR comparison—will validate the results of our proof of concept study, then our technique will be ready to application in the clinical arena (23).

## Data Availability

The raw data supporting the conclusions of this article will be made available by the authors, without undue reservation.
